# Metagenomics of microbiota following probiotic supplementation in rats subjected to intestinal anastomosis^☆^

**DOI:** 10.1016/j.sopen.2023.06.007

**Published:** 2023-06-28

**Authors:** Tiago Jacometo Coelho de Castilho, Gustavo Henrique Doná Rodrigues de Almeida, Eneri Vieira de S.L. Mello, Antônio Carlos L. Campos

**Affiliations:** a Postgraduate Program in Surgical Clinic, Health Sciences Sector, Federal University of Paraná, Curitiba, PR, Brazil; b Animal Histotechnical Laboratory, Department of Morphophysiological Sciences, State University of Maringá, Maringá, PR, Brazil

**Keywords:** Microbiota, *Lactobacillus*, Colorectal surgery, Probiotics, Metagenomics

## Abstract

**Background:**

The use of probiotics positively modifies the composition and function of the intestinal flora, decreasing inflammation, and these changes improve the quality of intestinal anastomosis. Therefore, the objective of this study was to evaluate the metagenomics of the microbial community after probiotic supplementation in rats subjected to intestinal anastomosis.

**Methods:**

The probiotic chosen for this study was composed of the strains *Lactobacillus paracasei* LPC37, *Bifidobacterium lactis* HN0019, *Lactobacillus rhamnosus* HN001 and *Lactobacillus acidophilus* NCFM. Both groups underwent two colostomies, one in the right colon and the second in the rectosigmoid colon, followed by anastomosis with eight interrupted stitches. The rats were killed on the fifth day of PO. Changes in the intestinal microbiota were evaluated by means of a metagenomic study that evaluated bacterial alpha and beta diversity indices.

**Results:**

Although there were no significant differences for any alpha diversity index, changes were observed for beta diversity indexes in the microbiota of rats. The group that received the probiotic preserved and even increased the abundance of beneficial bacterial genera and, at the same time, decreased the abundance of potentially pathogenic bacteria, promoting a favorable environment for anastomoses' healing.

**Conclusion:**

The use of probiotics had a positive impact on the quality of the intestinal microbiota.

## Highlights

• The probiotic supplementation promoted structural changes in the intestinal microbiota.
• Beneficial bacterial genera were preserved and increased in abundance.
• Moreover, potentially pathogenic bacteria abundance decreased.

## Introduction

The intestinal microbiota plays an important role in balancing the immune system through complex interactions between resident bacterial species and the inflammatory response. Several intestinal bacterial species promote cell immunity and stimulate the production of immune cells, improving overall immune functions. Therapeutic methods of gut microbiota modification in colorectal cancer management–fecal microbiota transplantation, prebiotics, probiotics, and synbiotics [1]. The microbial composition of the intestinal tract is often stable under physiological conditions and consists mainly of commensal bacteria [2]. These bacteria are essential for the normal function of the gastrointestinal tract (GIT), producing vitamins, short-chain fatty acids and other important organic molecules [[3], [4], [5]].

However, several processes can induce changes in the local microbiota, transforming commensal microorganisms into aggressive pathogens [2,6]. In particular, during intestinal surgery, pathogenic bacteria can degrade the injured tissue, causing an interruption of the healing cascade that leads to dehiscence and consequent leakage through the anastomotic line [7]. For example, cancer patients who underwent colorectal resection and subsequent anastomosis demonstrated significant variations in the intestinal microenvironment, including increased pathogenic bacterial species [8].

Therefore, the microbiota is important in the healing and recovery process of patients who receive anastomotic surgery in the GIT [9]. Under physiological conditions, there is a balance of the resident microbiota that is determined by complex and highly regulated cell interactions. Dysbiosis caused by surgical procedures leads to an altered intestinal environment, with impaired barrier and immune functions [7]. Therefore, modulation of the local microbiota through the use of probiotics has been used to reverse dysbiosis caused by anastomotic surgery [10].

Probiotics are living microorganisms that can be used to positively modulate the intestinal microbiota by activating the immune system, inhibiting pathogenic strains, and stimulating the secretion of immunoglobulins [11]. Therefore, the use of probiotics in patients undergoing abdominal surgical procedures can promote significant improvements in patient recovery by reducing infectious complications and overall improvements in surgical results [12]. Clinically, the use of probiotics to modulate the GIT microbiota after anastomosis reduces the total length of hospital stay, the number of days of ventilatory support, and days in intensive care [10,13,14]. Given these benefits and the complications that can follow GIT surgery, the objective of this study was to evaluate the modulating effect of probiotics on the intestinal microbiota after colonic transection and anastomosis in rats.

## Methods

In this experimental study, 36 adult male Wistar rats (*Rattus norvegicus albinus*, Rodentia, Mammalia), with body weight ranging from 220 to 320 g, were used. Animals were housed individually in boxes receiving *ad libitum* water and rat food (Nuvilab CR1®). The experiment was in accordance with the ARRIVE guidelines and was approved by the Maringá State University Animal Use Ethics Committee under the protocol number CEUA 5684270616.

After initial acclimatization, the probiotic study group (*n* = 18) received rat food *ad libitum* for two weeks. In the week preceding the surgical procedure (preoperative seven-day period) and in the postoperative period (five-day period), the rats in the study group received oral probiotic supplementation at a dose of 250 mg/day. The control group (*n* = 18) also received *ad libitum* rat food for 14 days. In the week before (7 days) the surgical procedure, the control group received a formula of maltodextrin at a dose of 250 mg/day and for additional five days after the surgical procedure, which was isocaloric and isovolumetric to the diet of the study group. The administration of probiotics or placebo was performed orally using a spatula with a dosimeter containing the appropriate dose of probiotic or maltodextrin administered directly (oral). The probiotic formula used was Probiatop® (Healthy Functional Nutrition São Paulo, Brasil), containing the strains *Lactobacillus paracasei* LPC37, *Bifidobacterium lactis* HN0019, *Lactobacillus rhamnosus* HN001 and *Lactobacillus acidophilus* NCFM (doses 1 × 109 CFU/g).

The surgical procedure, including the preoperative and postoperative periods, is described in detail [15]. Briefly, both groups underwent two colostomies, one in the right colon and the other at the rectosigmoid junction, followed by reanastomosis with eight interrupted 6-0 mononylon stitches. All rats were killed on the fifth postoperative day. The periods established for sample collection were the day of surgery (pre-surgical period - D7) and the day of sacrifice (post-surgical period - D12). On those days, feces were collected directly from the intestine through the continuity solution created between the anastomoses of the proximal and distal (rectum) segments of the large intestine. For evaluation, 40 samples were used, with 20 samples collected on the day of surgery (10 samples from the control group and 10 samples from the study group) and another 20 samples on the day of sacrifice (10 samples from the control group and 10 samples from the study group) totalling 10 samples from the control group and 10 samples from the study group at each moment.

The metagenomic study began with DNA genome sequencing of DNA. The total genome DNA of the samples was extracted using the CTAB/SDS method. First, DNA concentration and purity were monitored on a 1 % agarose gel. Depending on concentration, DNA was diluted to 1 ng/μL with sterile water. For amplifier generation, 16S rRNA/18SrRNA/ITS genes from distinct regions (16SV4/16SV3/16SV3-V4/16SV4-V5, 18S V4/18S V9, ITS1/ITS2, Arc V4) were amplified with specific primers. All PCR reactions were performed with Phusion® high-fidelity PCR Master Mix (New England Biolabs, Ipswich, USA). For the quantification and qualification of the PCR products, the same volume of loading buffer (contained in SYB green) was mixed with the PCR products and a 2 % agarose gel electrophoresis was performed for detection. Samples with a bright main band between 400 and 450 bp were chosen for further experiments. Library preparation and sequencing were generated using the TruSeq® DNA PCR-free sample preparation kit (Illumina, San Diego, USA) following the manufacturer's recommendations. Library quality was evaluated on the Qubit @ 2.0 fluorometer (Thermo Scientific, Waltham, USA) and on the Agilent Bioanalyzer 2100 system. Finally, the library was sequenced on an IlluminaHiSeq2500 platform, and 250-bp paired reads were generated.

Alpha-diversity indices, including richness (Observed, Chao1, ACE) and diversity (Shannon, Simpson, InvSimpson, Fisher, Evennes), and beta-diversity indices (Heatmap and Principal Coordinate Analysis) were calculated using Phyloseq version 1.22.3. All data were normalised to the median. To test whether there were statistical differences between the richness and diversity indices of control and probiotic, the Wilcoxon test was used. This test was selected for being a nonparametric test, so it does not depend on the normality of the data, as sequencing data tend to have a nonnormal distribution. Identification of the differentially abundant genus was performed with the DESeq2 version 1.18.1 software package, with bacteria considered differentially abundant at a *p*-value <0.05 and |*logFoldChanged*| > 2.

## Results

### Anastomosis

The anastomosis success was evaluated histologically through hematoxylin-eosin and picrosirius red tests as well as through tensiometry [15]. The value of the maximum traction force (FMT) was 1.2 ± 0.2 N in the control group and 1.5 ± 0.3 N (*p* = 0.0250 95.27 % CI) in the probiotic group. The maximum traction force (MTF) of the control and probiotic groups were, respectively, 1.1 ± 0.2 N and 1.4 ± 0.3 N (*p* = 0.0116 95.27 % CI). Thus, we verified the positive impact of using the probiotic on the anastomosis tensile strength [15].

Post-operative complications included an intestinal fistula in the rectosigmoid anastomosis in four animals (22.22 %) in the control group and two (11.11 %) (*p* = 0.6581) in the probiotic group. The animals that presented fistula died.

### Alpha diversity indices

**Fig. 1:**
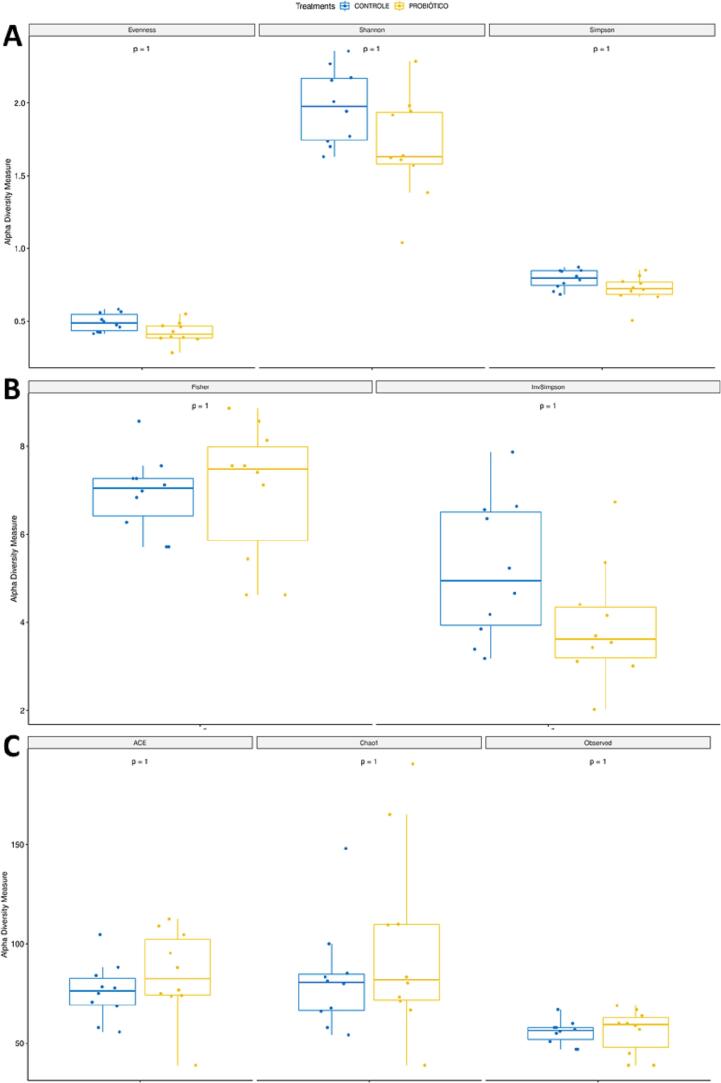
Alpha diversity indices evaluating the richness and diversity in the gut microbiota of the preoperative period. A = Evennes, Shannon, and Simpson, B = Fisher and InvSimpsom, C = Chao1, ACE and Observed.

**Fig. 2:**
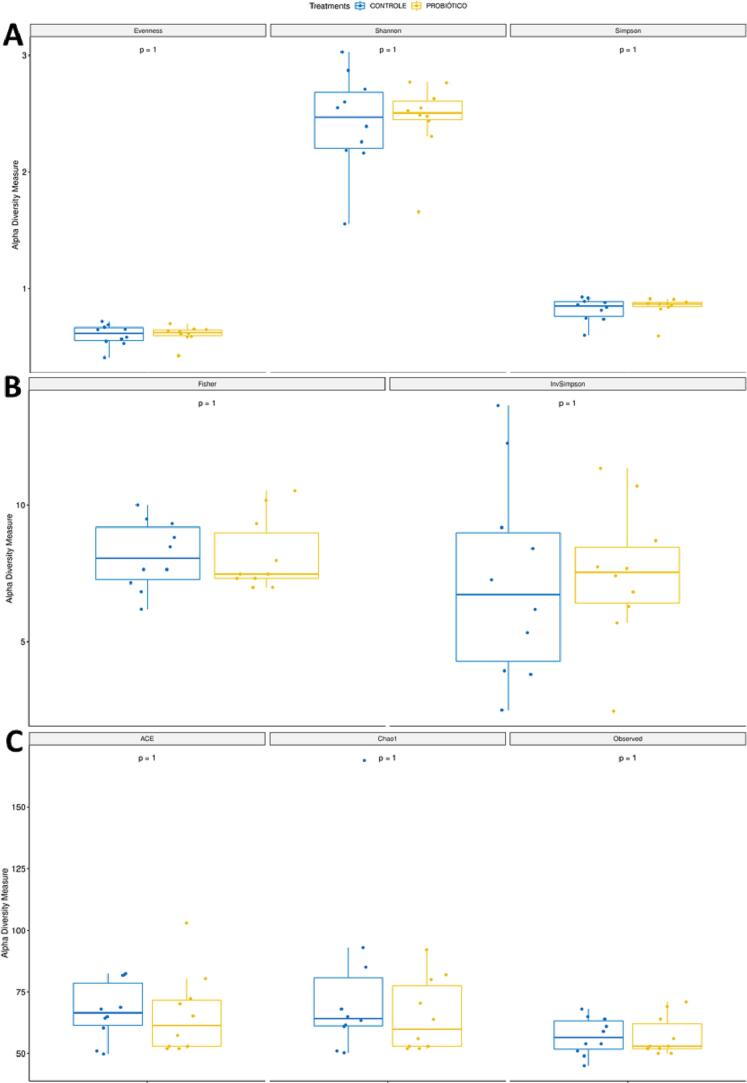
Alpha diversity indices evaluating the richness and diversity in the intestinal microbiota of the postoperative period. A = Evennes, Shannon, and Simpson, B = Fisher and InvSimpsom, C = Chao1, ACE and Observed.

Alpha diversity indices describe the variation of species within a community or sample. There were no significant differences (*p* = 1) for all alpha diversity indices that evaluated the richness and diversity of the gut microbiota (Evennes, Shannon, Simpson, Fisher, InvSimpsom, Chao1, ACE and Observed) between the control and probiotic group for both preoperative (Fig. 1ABC) and postoperative periods (Fig. 2ABC).

### Beta-diversity indexes

**Fig. 3:**
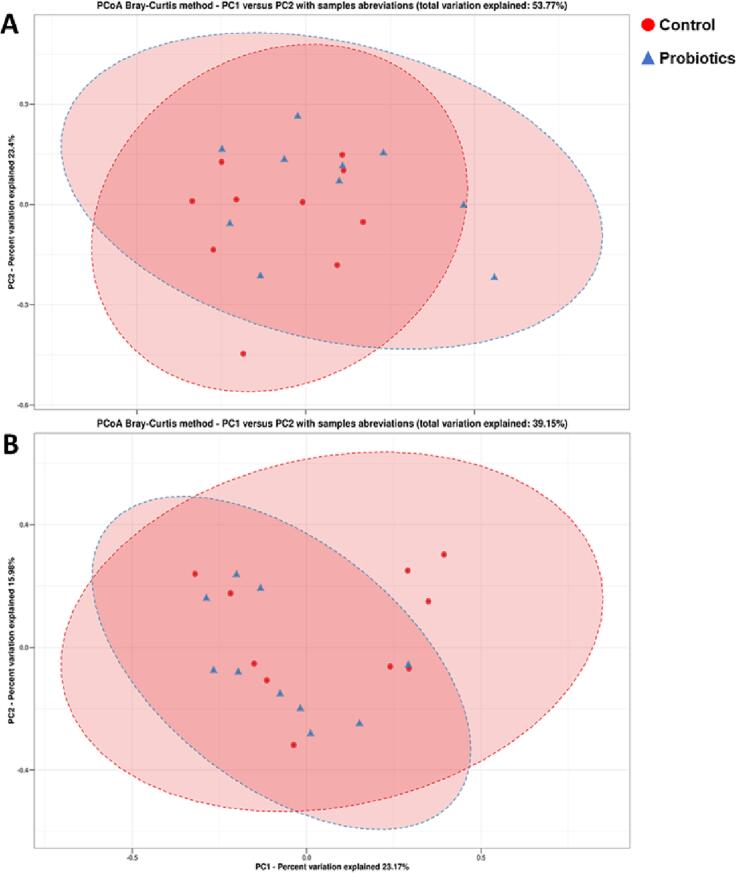
Principal Coordinate Analysis (PCoA) showing the clustering profile between the control and probiotic groups according to microbial community composition for the preoperative (A) post-operative (B) periods. The percentage of variability explained by the corresponding coordinate is indicated on the axis. Each point represents a sample, red symbols indicate the control, and blue symbols indicate the treatment of probiotics. Ellipses serve as a visual guide to group differences. Please note that the closer the distance between different samples, the more similarities exist in the species composition. Samples with high similarity of community structure are incline to be clustered together, while communities with large variation will be separated remotely on the plot area. (For interpretation of the references to colour in this figure legend, the reader is referred to the web version of this article.)

Beta diversity indices assess the variation in composition between different communities of bacterial species. Principal Coordinate Analysys (PCoA) plots based on Bray-Curtis methods were generated to visualise differences in the structure of the microbial community at the OTU level (Fig. 3), which in general explained 53.77 % for the preoperative period (Fig. 3A) and 39.15 % for the postoperative period (Fig. 3B). The PCoA plots did not provide a clear separation of groups, and therefore no significant differences in the microbial community were observed for both the preoperative and postoperative periods (Fig. 3AB).

Together, 111 genera and 243 species of bacteria that make up the control and probiotic groups were identified. Differential abundance analysis was performed using the DESeq2 method, which estimates the mean variance of high-throughput sequencing assays and tests differential expression based on a model using the negative binomial distribution. For the preoperative period, the two genera that were differentially abundant, *Ruminiclostridium* and *Fusicatenibacter*, decreased significantly in the probiotic group compared to the control. For the postoperative period, only the *Collinsella* genus was identified as differentially abundant, increasing significantly in the probiotics group compared to the controls.

**Fig. 4:**
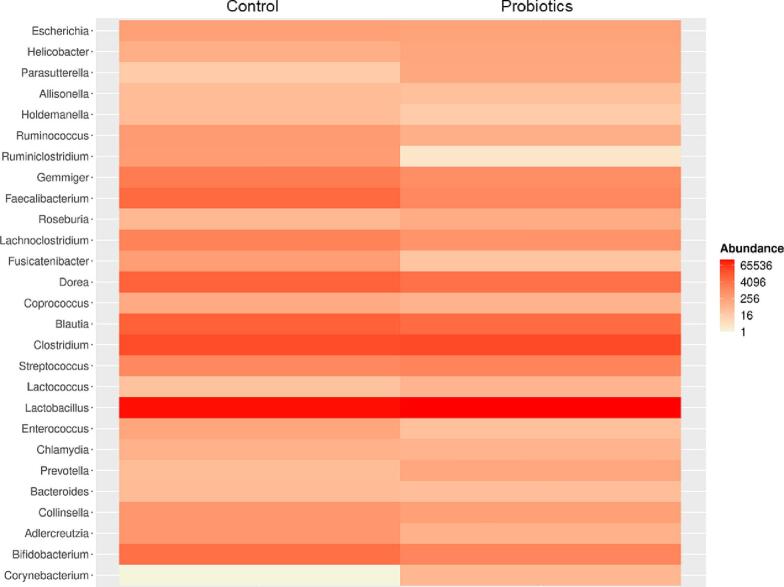
Heat map comparing the frequency of differential abundance of bacterial genera between the control and probiotic group in the preoperative period.

**Fig. 5:**
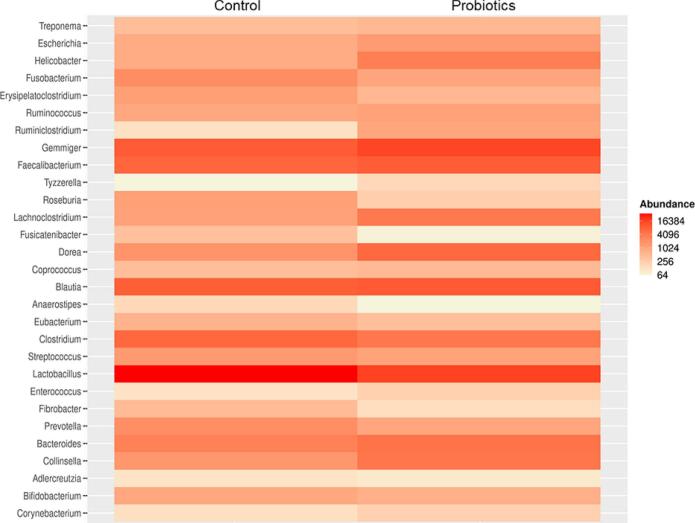
Heat map comparing the frequency of differential abundance of bacterial genera between the control and probiotic group in the preoperative period.

Furthermore, to avoid misrepresentation of important taxonomic groups that were not detected by the DESeq2 method, the 50 most abundant bacterial species in treatments were evaluated. Therefore, all genera identified in the differential abundance analysis are represented in the heat maps (Fig. 4, Fig. 5). This allows us to focus only on taxa that respond to some disturbance inserted into the analysed environment. However, before building the heat maps, OTU's with counts <10 and that were not present in at least 20 % of the samples were eliminated from the heat maps.

The heat map in Fig. 4 detected differences in the differential abundance of 27 bacterial genera between the control and treatment groups for the preoperative period. Individually, the most abundant bacterial genera for both control and probiotics remained unchanged, being composed of the genera *Lactobacillus*, *Clostridium*, *Blautia*, *Dorea*, and *Feacaliumbacterium*, all belonging to the Firmicutes phylum (Fig. 4). In comparison, the *Lactobacillus* genus was the most abundant for both the control group and the probiotic group. For the *Clostridium* genus, there was an increase in abundance for the control group compared to the probiotics group. However, for the genera *Blautia*, *Dorea*, and *Feacaliumbacterium*, there was a decrease in abundance for the group that received probiotic treatment compared to the control group. The genera *Bifidobacterium* and *Adlercreutzia*, belonging to the phylum Actinobacterium, also showed a decrease in abundance in the treatment group compared to the control group. The other identified bacterial genera were significantly lower in abundance in both groups (Fig. 4).

The heat map in Fig. 5 showed the abundance of 29 bacterial genera between control and treatment, for the postoperative period. Individually, the most abundant bacterial genera for the control group were *Lactobacillus*, Geminger *Clostridium*, *Blautia*, *Dorea*, and *Feacaliumbacterium* (Fig. 5). For the probiotic group, the most abundant genera were *Lactobacillus*, *Geminger Clostridium*, *Blautia*, *Dorea*, *Feacaliumbacterium*, *Bacteriodes*, and *Laminiclostridium* (Fig. 5). *Lactobacillus* was the most abundant genus for both periods; however, there was a decrease in abundance in the probiotic group compared with the control group. The genera *Clostridium*, *Pravotela Streptococcus*, and *Enterococcus* also decreased in abundance in the probiotics group (Fig. 5). For the genera *Geminger Clostridium*, *Blautia*, *Dorea*, *Feacaliumbacterium*, and *Bacteriodes*, there was an increase in abundance in the probiotic group as compared to the control group (Fig. 5).

## Discussion

In the present study, we sought to evaluate the modulating effect of probiotics on the intestinal microbiome after colonic surgery and anastomosis in rats. As shown by the results of the metagenomic analysis, the intestinal microbiota underwent structural changes, mainly in terms of relative abundance, but not in species composition. The alpha diversity index denotes a mathematical measure of species diversity in a community, and beta diversity means differential abundance in the microbial community between different environments. Although there was no statistical significance for alpha diversity, there was a large decrease in the overall relative abundance (beta diversity) of bacterial genera in the postoperative period compared to the preoperative group, both for the control and probiotic treatment.

For example, the most abundant genera *Lactobacillus* had a decrease of approximately 75 % between the preoperative and postoperative periods. However, independently, the most abundant genera remained unchanged between the control and treatment groups for each period. In general, within all samples, the most abundant genera were *Lactobacillus*, *Blautia*, *Dorea*, *Feacaliumbacterium*, *Gemminger*, *Collinsella*, *Bacteriodes*, *Bifidobacterium*, *Clostridium*, *Pravotella*, *Streptococcus*, *Enterococcus*, and *Escherichia*. *Lactobacillus* was the most abundant genus, while the genera *Clostridium*, *Pravotella*, *Streptococcus*, *Enterococcus* decreased in abundance, and the genera *Geminger*, *Clostridium*, *Blautia*, *Dorea*, *Feacaliumbacterium* and *Bacteriodes* increased in abundance in the probiotics group compared to the control group, particularly in the postoperative period.

Bacterial metabolic products are important for GIT. Butyrate, for example, is the main source of energy for colonic cells. Therefore, microbial dysbiosis, characterized by a decrease and a change in microbial composition and activity, can contribute to the onset of various colonic disorders, including cancer [7]. Furthermore, dysbiosis also consists of changes in microbial metabolism, which consequently alters the release of metabolites, such as small chain fatty acids, bile acids, phenolic compounds, lipopolysaccharide and peptidoglycans, and bacterial compounds that act on the essential functions of the host's intestine, including endocrine and immunity functions, and also barrier function and epithelial repair [16]. Therefore, there is a strong correlation between the different microbial communities in the final repair of the wound, in the regenerative process of intestinal epithelial cells and in inflammatory processes [9]. When the intestines are damaged by anastomotic surgery, the composition of native microflora changes and pathogenic microorganisms can overgrow, leading to dehiscence and anastomotic leakage [17,18].

A recent meta-analysis with randomized controlled trials evaluated the effect of probiotics or symbiotics in adult patients who underwent elective colorectal, upper gastrointestinal, transplant, and hepatopancreaticobiliary surgeries. A total of 34 randomized controlled trials reporting on 2723 participants were included. In the intervention arm, 1354 patients received probiotic or symbiotic preparations, while 1359 patients in the control arm received placebo or standard care. Perioperative administration of probiotics or symbiotics significantly reduced the risk of infectious complications after abdominal surgery [relative risk (RR) 0.56; 95 % confidence interval (CI) 0.46–0.69; *P* < 0.00001, *n* = 2723, *I*^2^ = 42 %] [10].

Therefore, probiotics have been used to regulate and increase bacterial diversity in GIT. Probiotics have the potential to decrease the severity of intestinal mucositis injury by reducing the secretion of pro-inflammatory cytokines, by releasing anti-inflammatory cytokines, maintaining mucin secretion, and preventing apoptosis of epithelial cells due to oxidative damage [19]. One of the mechanisms also identified in the use of probiotics is the inhibition of pathogenic enteric bacteria in the intestinal tract. Another important mechanism inherent in probiotics is the increase in epithelial barrier and immunoregulation functions. For example, species of the genus *Lactobacillus* have an impact on eliminating pathogenic bacteria and maintaining intestinal permeability in the injured intestine [17].

The decrease in the total abundance of bacteria present in the GTI may be an effect of tissue ischemia during the surgical procedure. Mesenteric ischemia and subsequent reperfusion can induce a decrease in *Lactobacillus* bacteria in the ileum and colon [18], and a significant reduction in the total number of beneficial dominant obligate anaerobic bacteria [20]. Furthermore, unlike this study, there was also a significant increase in the number of potentially pathogenic genera *Enterobacteriaceae*, *Staphylococcus*, *Pseudomonas* and *Clostridium* after colorectal surgery. This increase in pathogenic bacteria can reach up to 500 times for *Enterococcus* and 200 times for *Eschericaea* after a surgical procedure in animal models [21]. A similar change was demonstrated in patients undergoing colectomy, with a significant increase in the *Enterococcus*, *Staphylococcus*, and *Pseudomonas* genera [22].

The reduction in pathogenic genera observed in this study can be attributed to the increase in the *genus Bacteroides*. These microorganisms participate in intestinal microbial regulation through the production of bacteriocins. Bacteriocins are proteins or peptides that have a broad-spectrum bactericidal effect against microorganisms, providing a selective advantage by eliminating microbial competitors. They can play a defensive role, acting to inhibit the invasion of bacterial strains or species occupying a specific niche and limiting the advance of colonization of neighboring cells [23]. Thus, in this study, the group that received probiotic supplementation showed a decrease in the pathogenic genera *Clostridium*, *Pravotella*, and *Streptococcus* while the genera *Enterococcus* and *Escherichia* remained unchanged. Furthermore, in an animal model study, the genus *Bacteriodes* demonstrated an important role in the reconstruction of the mesenchymal microvascular network, stimulating angiogenesis and thus also decreasing the appearance of anastomotic fistulas [24]. Additionally, mesenteric ischemia and subsequent reperfusion can induce a decrease in *Lactobacillus* bacteria in the ileum and colon [18], and a significant reduction in the total number of beneficial dominant obligate anaerobic bacteria in animal models [20]. There was also a significant increase in the number of potentially pathogenic genera *Enterobacteriaceae*, *Staphylococcus*, *Pseudomonas*, and *Clostridium* after colorectal surgery. This increase in pathogenic bacteria can reach up to 500 times for *Enterococcus* and 200 times for *Eschericaea* after a surgical procedure in animal models [21].

Two other beneficial bacterial genera, *Dorea* and *Blautia*, also increased postoperatively with the use of probiotics compared to controls. These bacteria exhibit anti-inflammatory functions depending on the surrounding intestinal bacteria and or the available nutrients. There is a synergy between these bacterial genera, as *Blautia* uses gases produced by *Dorea* to promote its growth [25]. Together, these two genera are hypothesised to influence intestinal barrier function [25]. The barrier function promoted by the microbiota has a strong relationship with the healing process of intestinal anastomosis. The function of the intestinal barrier regulates host transport and defense mechanisms, since tolerance to commensal bacteria and immunity against pathogens depend on recognition, processing, and response mechanisms through epithelial cells [26]. Similar to the *Bacteroides* genus, the *Blautia* genus has the ability to produce bacteriocins that inhibit the colonization of pathogenic bacteria in the intestine, affecting the composition of the intestinal microbiota [27].

The main component of probiotic preparations is the species of the *Lactobacillus* genus in this and other studies, and despite the fact that the *Lactobacillus* genus suffered a decrease in the postoperative period, this genus was still the most abundant in our study. The genus *Lactobacillus* promotes beneficial effects on wound healing by stimulating restitutive signaling and increasing the migration and proliferation of epithelial cells [16]. One mechanism by which lactobacillus-containing probiotics exert beneficial effects appears to be by improving chronic systemic inflammation. For example, *Lactobacillus* strains have been shown to reduce concentrations of bacterial-derived endotoxins, lipopolysaccharides, and related inflammatory factors [28].

Previously, mice fed the probiotic used in this study (Probiatop®, *Lactobacillus paracasei*, *Bifidobacterium lactis*, *Lactobacillus rhamnosus* and *Lactobacillus acidophilus*) had a reduction of 50 % in the appearance of anastomotic dehiscence [15]. Additionally, patients have also benefitted from probiotic supplementation. The use of probiotics in intestinal surgery reduces the risk of infections, with fewer days of hospital stay, intestinal dysbiosis, and non-infectious complications [29,30]. Similarly, patients undergoing colorectal cancer surgery with probiotic supplementation exhibited a significantly lower rate of all major postoperative complications, surgical site infections, and anastomotic leakage [13]. Furthermore, symbiotic formulations that combine fructooligosaccharides with *Lactobacillus acidophilus*, *Lactobacillus paracasei*, *Lactobacillus rhamnosus* and *Bifidobacterium lactis* for cancer patients subjected to colon resection was associated with lower levels of inflammatory markers, lower rate of surgical complications, less use of antibiotics, and no mortalities associated with the surgical procedure [14].

Finally, this study has demonstrated the modulating effects of probiotic supplementation on the intestinal microbiota after colonic surgery and subsequent anastomosis. There was a decrease in the potentially pathogenic genera *Clostridium*, *Pravotella*, *Streptococcus*, and *Enterococus*, and, in contrast, there was an increase in the beneficial bacterial genera *Lactobacillus*, *Bacterioides*, *Blauta* and *Dorea* in rats supplemented with probiotics. Understanding intestinal microbiota shifts, it is possible to target therapeutic treatments with probiotics that allow improved healing of anastomosis after resection surgery. However, it is necessary to investigate the possible advantages of this modulating effect in patients undergoing colorectal surgery to further understand the beneficial role of probiotics in preventing postoperative complications.

## CRediT authorship contribution statement

Tiago Castilho: Conceptualization; Data acquisition; Formal analysis; Investigation; Methodology; Writing - original draft; Writing - review & editing. Gustavo Almeida: Conceptualization; Data acquisition; Formal analysis; Methodology; Writing - original draft. Eneri Mello: Conceptualization; Methodology; Writing - original draft; Writing - review & editing. Antonio Campos: Conceptualization; Data acquisition; Formal analysis; Investigation; Methodology; Writing - original draft; Writing - review & editing.

## Funding sources

This research did not receive any specific grant from funding agencies in the public, commercial, or non-profit sectors.

## Ethics approval

This work was approved by the Maringá State University Animal Use Ethics Committee under the protocol number CEUA 5684270616.

## Declaration of competing interest

All authors declare that they have no conflict of interest.
